# Probabilistic method for detecting copy number variation in a fetal genome using maternal plasma sequencing

**DOI:** 10.1093/bioinformatics/btu292

**Published:** 2014-06-11

**Authors:** Ladislav Rampášek, Aryan Arbabi, Michael Brudno

**Affiliations:** ^1^Department of Computer Science, University of Toronto, Toronto M5S 2E4, ^2^Centre for Computational Medicine and ^3^Genetics and Genome Biology, Hospital for Sick Children, Toronto M5G 1L7, Canada

## Abstract

**Motivation:** The past several years have seen the development of methodologies to identify genomic variation within a fetus through the non-invasive sequencing of maternal blood plasma. These methods are based on the observation that maternal plasma contains a fraction of DNA (typically 5–15%) originating from the fetus, and such methodologies have already been used for the detection of whole-chromosome events (aneuploidies), and to a more limited extent for smaller (typically several megabases long) copy number variants (CNVs).

**Results:** Here we present a probabilistic method for non-invasive analysis of *de novo* CNVs in fetal genome based on maternal plasma sequencing. Our novel method combines three types of information within a unified Hidden Markov Model: the imbalance of allelic ratios at SNP positions, the use of parental genotypes to phase nearby SNPs and depth of coverage to better differentiate between various types of CNVs and improve precision. Our simulation results, based on *in silico* introduction of novel CNVs into plasma samples with 13% fetal DNA concentration, demonstrate a sensitivity of 90% for CNVs >400 kb (with 13 calls in an unaffected genome), and 40% for 50–400 kb CNVs (with 108 calls in an unaffected genome).

**Availability and implementation:** Implementation of our model and data simulation method is available at http://github.com/compbio-UofT/fCNV.

**Contact:** brudno@cs.toronto.edu

## 1 INTRODUCTION

Until recently, the prenatal analysis of a fetal genome required samples directly obtained from the fetus by invasive procedures like chorionic villus sampling or amniocentesis, where amniotic fluid is sampled from around the developing fetus. Amniocentesis, however, has several important disadvantages. Foremost, it carries a non-trivial risk of miscarriage [estimated procedure-related fetal loss rate is 0.6–1% (Douglas *et al.*, 2007)], and hence is refused by a fraction of patients. Secondly, amniocentesis cannot be performed too early, as the risk of miscarriage rises significantly, and is typically indicated for the 15th week of pregnancy, outside of the time-frame for the safest abortion options (<12 weeks) and leaving only limited time for follow-up analysis. Finally, amniocentesis is a complex and expensive medical procedure ($1500–$3000). Consequently, amniocentesis is typically performed only to confirm or reject a diagnosis if a genetic disease is suspected, e.g. high likelihood of Down syndrome based on prenatal ultrasound.

The last several years has seen the initial development of alternative, non-invasive methods for prenatal genetic testing. Prominent among these are methods that are based on analysis (arrays or sequencing) of cell-free DNA (cfDNA) extracted from maternal blood plasma, which contains an admixture of fetal and maternal DNA. The fraction of fetal DNA in such an admixture varies depending on multiple factors, including maternal weight and size of the fetus, but typically builds up from ∼5–7% early in the pregnancy to 10% at week 10 (Wang *et al.*, 2013) to as much as 50% before delivery (Fan *et al.*, 2012; Wang *et al.*, 2013). In experiments conducted by Kitzman *et al.* (2012) (and utilized in this article), the estimated admixture in samples obtained at 8 weeks and 18.5 weeks of gestation was 7 and 13%, respectively.

The decreasing cost of DNA sequencing has made it practical to directly sequence cfDNA extracted from maternal blood to identify likely genetic disorders present in the fetus. Non-invasive methods are becoming more commonly used to directly identify aneuploidies (abnormal chromosome counts) and are also enabling preventive screening for heritable genetic diseases, resulting in better prenatal health care (Saunders *et al.*, 2012). While most non-invasive genetic diagnostics aim to test for a particular previously known biomarker, Kitzman *et al.* (2012) demonstrated the possibility of the reconstruction of the whole genome of the fetus by combining whole-genome sequencing of parental genomes with deep sequencing of cfDNA from maternal plasma (78× coverage). The key intuition in this method is the comparison of allelic ratios at individual SNP loci, as the inheritance of a particular paternal allele affects the percentage of reads with that allele at the particular position in the genome. This method heavily relies on the availability of phased parental genotypes, as these allow for the inference of likely co-inherited SNPs, leading to an improvement in the signal-to-noise ratio. It consequently provides for high accuracy identification of inherited (98% accuracy) but not *de novo* single nucleotide variants (17 correct calls out of 44 true *de novo* sites, with 3884 called positions).

While most efforts to detect copy number variants (CNVs) from cfDNA sequencing have so far concentrated on whole-chromosome events [e.g. Chu *et al.* (2009)], the past year has seen the first few attempts at methods for genome-wide identification of sub-chromosomal *de novo* CNVs. Such methods are desired to enable non-invasive prenatal screening for diseases like DiGeorge syndrome (∼3 Mb deletion), Prader-Willi syndrome (the deletion subtype caused by a ∼4 Mb deletion) and other disorders associated with a mid to large sized CNV. So far two manuscripts address the problem of detecting sub-chromosomal CNVs (Chen *et al.*, 2013; Srinivasan *et al.*, 2013). While the exact methods used in both of these approaches differ, both rely on depth of coverage: they map the reads to the genome, divide the genome into bins and identify the CNVs by comparing the number of reads mapped to each bin. The key idea in these methods is that deletions/duplications will result in more/fewer fetal reads within a window, and this difference can be identified using statistical methods. Srinivasan *et al.* (2013) use depth-of-coverage computed in 1 Mb windows across the genome to identify CNVs that are typically >1 MB, though they do report discovery of a 300 kb CNV. Nine of the 22 discovered CNVs in 11 patients were concordant with karyotyping results, with most discrepancies being short (<1 Mb) CNVs. Importantly, they use extremely short (25 bp) reads, allowing for larger number of fragments at equal coverage depth. Chen *et al.* (2013) use even larger 10 Mb windows, again considering only the number of fragments mapped, and are able to successfully identify variants 9–29 Mb with only one false positive among six true positives in 1311 patients. Both methods utilize low coverage WGS of the plasma cfDNA, while leveraging the large number of samples.

In this manuscript, we introduce a novel model for non-invasive prenatal identification of *de novo* CNVs with increased sensitivity compared with methods published so far. Our method combines three types of information within a unified probabilistic model. First, our method takes advantage of the imbalance of allelic ratios at SNP positions that are introduced by various types of paternally and maternally inherited CNVs. Secondly, following the work of Kitzman *et al.* (2012), we use parental genotypes to phase nearby SNPs, modelling their co-inheritance (or recombination) and thus improving the signal-to-noise ratio. Finally, we observed that allelic ratios poorly differentiate between certain types of CNVs: for example, as further described below, a duplication of a paternally inherited allele results in extremely similar allelic ratios to deletion of a maternally inherited one. We thus combine the allelic ratios with the depth-of-coverage signal to better differentiate between such cases. Our simulation results, based on *in silico* introduction of novel CNVs into plasma samples with 13% fetal DNA concentration, demonstrate a sensitivity of 90% for CNVs >400 kb (with 13 calls in an unaffected genome), and 40% for 50–400 kb CNVs (with 108 calls in an unaffected genome).

## 2 METHODS

Our method models two types of signal from the data: (i) imbalance of the allele distributions at SNP loci (discussed in Section 2.1) and (ii) number of fragments sequenced from ∼1 kb genomic regions (discussed in Section 2.2). Though each of these is noisy, the two are (nearly) independent (modulo number of reads overlapping the SNP position) variables and can be combined into a single generative model. For this purpose, we use a Hidden Markov Model (HMM), where we interpret the allele counts at SNP loci as emissions, while the coverage is used as a prior probability for each state (see Section 2.3).

For our method, we assume that we have phased haplotypes of both parents, and deep sequencing data of cfDNA from maternal plasma. In practice we used whole-genome sequencing (WGS) data for the parents, with phasing based on 1000 Genomes data (see Section 3.1). All *de novo* CNVs thus correspond to a particular parental haplotype duplication or deletion event. Labelling the two maternal and paternal haplotypes as *M_A_*, *M_B_*, *P_A_*, *P_B_*. For each inheritance pattern—normal inheritance, maternal duplication, paternal duplication, maternal deletion, paternal deletion—we introduce a set of *phased inheritance patterns* that enumerates all the possible configurations of fetal haplotypes corresponding to the respective inheritance pattern. For example a duplication in the maternal gamete will consist of one (or more) of six phased inheritance patterns:

$$\begin{array}{l} M_{A}M_{A}P_{A},M_{A}M_{B}P_{A},M_{B}M_{B}P_{A},\\ M_{A}M_{A}P_{B},M_{A}M_{B}P_{B},M_{B}M_{B}P_{B} \end{array}$$

There are a total of 20 phased inheritance patterns (PP): six each for maternal/paternal duplication, two each for maternal/paternal deletion and four for normal inheritance). We refer to the number of alleles (copy count) inherited by the fetus as |PP|. We use *r* to refer to the percentage of cfDNA that is fetus-derived; this parameter is estimated from positions in the genome where the parents are homozygous for alternate alleles.

### 2.1 SNP allele distribution

For every SNP locus we observe a distribution of nucleotides in maternal plasma reads. In this section we focus on calculating the probability of the observation with respect to a phased inheritance pattern. Formally, we observe the counts of the four nucleotides $\left\{k_{A},k_{C},k_{G},k_{T}\right\}$ and compute the probability of observing each of these from a particular phased inheritance pattern PP. Ideally, these counts should follow multinomial distribution with the parameter vector $\left(p_{A},p_{C},p_{G},p_{T}\right)$. However we have found that modelling them as independent Gaussians with variance equal to the mean (as an approximation of the Poisson), makes the inference of the inheritance pattern more robust to noise.

More formally,

(1) $$Pr[k_{x}|M_{A},M_{B};P_{A},P_{B};r;PP]∼N\left(\mu_{x},\mu_{x}\right)$$

To compute the expected support $\mu_{x}$ for x∈{A,C,G,T}, we first adjust the mixture ratio *r* based on the expected number of fetal haplotypes |PP|, as absence/presence of an additional fetal copy in the plasma sample influences the local fetal mixture ratio. We accommodate this influence of |PP| expected fetal haplotypes instead of regular two as follows:

(2) $$r\prime =\frac{|PP|\cdot r/2}{\left|PP|\cdot r/2+(1-r\right)}$$

Then for each nucleotide *x* we compute the probability *p_x_* of observing a read supporting *x*. Such a read might have originated from multiple haplotypes, including two maternal haplotypes and |PP| fetal haplotypes. We can individually evaluate this probability for each haplotype and subsequently sum them to obtain *p_x_*:

(3) $$\begin{array}{l} p_{x}=\sum_{i\in \{A,B\}}\left[M_{i}\;\text{equals}\;x\right]\cdot m_{i}(1-r\prime )\\ +\sum_{y\in PP}\left[y\;\text{equals}\;x\right]\cdot \frac{r\prime }{\left|PP\right|} \end{array}$$

For reads putatively coming from maternal portion of the cfDNA sample, we correct for maternal CNVs by using the allele ratios *m_i_* as observed in maternal-only sequencing data. Additionally, to mitigate noise we add pseudocount α (proportional to the genome-wide coverage) to these counts.

(4) $$m_{i}=\frac{\alpha +\#\text{reads supporting}\;M_{i}\;\text{in maternal sample}}{2\alpha +\sum_{j\in \{A,B\}}\#\text{reads supporting}\;M_{j}\;\text{in maternal sample}}$$

We thus obtain the expected probability distribution for each nucleotide observed at this SNP locus.

To obtain the expected number of reads $\mu_{x}$ supporting particular variant at this SNP locus, we have to multiply *p_x_* by the number of reads mapped,

(5) $$\mu_{x}=p_{x}\cdot \#\text{mapped reads}$$

As we describe later, we use this probability distribution $N\left(\mu_{x},\mu_{x}\right)$ that is conditional on phased pattern PP as the emission distribution for each nucleotide in our HMM.

### 2.2 CNVs and depth of coverage

**Fig. 1.**
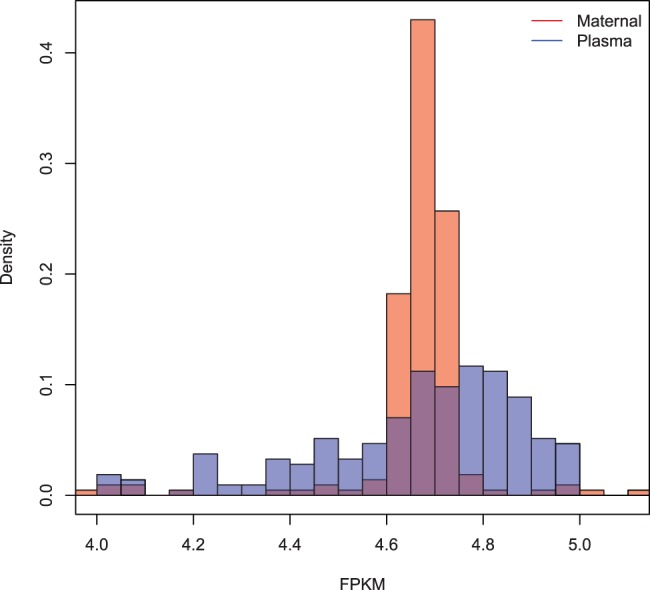
Distribution of fragments per kilobase of chromosome 1 per million fragments (FPKM) in 1 megabase segments for plasma sample (blue) and maternal sample (red) of the I1 trio

Variations in number of fragments sequenced per a region is a standard measure used for detection of mid- to large-sized CNVs [see Medvedev *et al.* (2009) for a review], and has also been used for CNV detection from maternal plasma (Chen *et al.*, 2013; Srinivasan *et al.*, 2013). However, the relatively low admixture of fetal DNA in the maternal plasma together with cfDNA sequencing biases considerably limit potential of methods relying on coverage signal from a single sample. Furthermore, the high variability of the coverage derived from blood plasma (Fig. 1) makes it difficult to identify shorter CNVs. Thus, methods Chen *et al.* (2013) and Srinivasan *et al.* (2013) use large bins and require multiple datasets to establish a baseline for CNV calling.

Simultaneously, the coverage forms an important complementary signal to the allelic distributions described above: certain ratios have very similar probability under different phased patterns, e.g. a deletion of a maternally inherited allele may yield distributions similar to a paternally inherited duplication. Incorporating the coverage signal helps to discriminate such states. In our method, we use the coverage information as a noisy predictor to complement the signal we obtain from SNP loci.

As a measure of coverage in a genomic region, we use *window ratio value* (WRV) analogous to the *bin ratio value* measure used by Srinivasan *et al.* (2013), which is essentially the number of fragments mapped to the region and normalized by the number of fragments mapped to other regions with similar GC content. Note that WRVs are independent of GC content and depth of sequencing of the sample.

For the purpose of our model, we split the genome to non-overlapping windows, each containing a single SNP, with breakpoints being in the middle between two adjacent SNPs. For each SNP *i* the corresponding $WRV_{i}$ for the window *W_i_* containing the *i*-th SNP position is then computed as the ratio of number of fragments $N_{W_{i}}$ mapped to *W_i_* to the sum of fragments mapped to 200 windows of the same size with GC content closest to *W_i_*:

(6) $$\begin{array}{ll} \mathrm{WR}V_{i} & =\frac{N_{W_{i}}}{\overset{}{\underset{W\in neigh_{\mathrm{GC}}^{200}(W_{i})}{\sum }}N_{W}} \end{array}$$

However, the variable length of the windows makes such computations expensive as computation of $neigh_{\mathrm{GC}}^{200}(W_{i})$ is linear in number of windows. To make the WRV computations practical, we scale $N_{W_{i}}$ to correspond to expected number of fragments as if $|W_{i}|=1$kb by multiplying $N_{W_{i}}$ by $1000/|W_{i}|$ (for clarity, not shown in our equations). Then WRVs in 1 kb bins can be precomputed, enabling us to find $neigh_{\mathrm{GC}}^{200}(W_{i})$ in time logarithmic from the number of bins. Using 1 kb bins is a good approximation as the mean distance between two adjacent SNP loci is expected to be 1 ∼ kb.

Overall, our goal is to estimate the probability of observing $WRV_{i}^{S}$ in the studied plasma sample conditional on the number of fetal haplotypes (|PP|), which is either three for duplication, one for deletion or two for normal inheritance. To do so, we use a reference sample to obtain $WRV_{i}^{R}$ for comparison (computed in the same genomic window *W_i_*). Further we need to compute two more reference $WRV_{i}^{R}$s, each scaled to reflect one CNV type. For duplication, we would expect to see (1+r/2) times more fragments while for deletion (1−r/2) times less fragments, thus the scaled $WRV_{i}^{R,|PP|}$ is estimated as

(7) $$\begin{array}{l} \mathrm{WR}V_{i}^{R,|\mathrm{PP}|}=\frac{N_{W_{i}^{R}}\cdot (1+\left(|\mathrm{PP}|-2\right)\cdot r/2)}{\overset{}{\underset{W\in neigh_{\mathrm{GC}}^{200}(W_{i}^{R})}{\sum }}N_{W^{R}}} \end{array}$$

Finally, we can compute the probability of $WRV_{i}^{S}$ being generated from an event with fetal allele copy count |PP| as:

(8) $$N(WRV_{i}^{R,|PP|}-WRV_{i}^{S};\mu =0,\sigma_{\text{noise}})$$

where we model the difference between $WRV_{i}^{S}$ and $WRV_{i}^{R}$ as a Gaussian noise with zero mean and empirically estimated variance $\sigma_{\text{noise}}$.

By normalizing the probabilities of $WRV_{i}^{S}$ w.r.t. all phased patterns, we obtain priors for each phased pattern that are used in the HMM described in the next section.

**Fig. 2.**
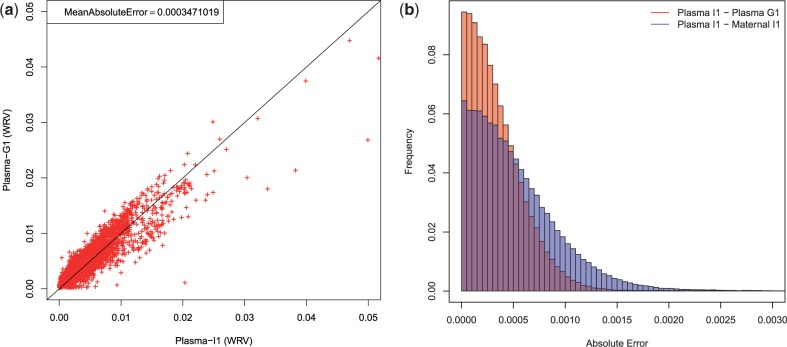
(**a**) A scatterplot demonstrating the correlation of WRV between plasma samples of I1 and G1 trios. The shown WRVs were computed for windows of size 1 kb in chromosome 1. (**b**) Histogram of absolute errors between WRVs from different samples, comparing distribution of absolute error between plasma samples of I1 and G1 trios (red), and between plasma sample and maternal sample of I1 trio (blue). There is a notably heavier tail in case of plasma to maternal sample error distribution, composed of windows with weak WRV correspondence—an artifact of wider coverage distribution in plasma cfDNA sample compared to standard WGS maternal sample (Fig. 1). This artifact causes plasma to maternal sample WRV comparison to have higher mean absolute error (0.000521, compared with 0.000347 for plasma I1 to plasma G1) even though they are from the same trio

As a reference plasma sequencing coverage, we use plasma sample of the G1 trio of Kitzman *et al.* (2012) dataset, as the overall coverages observed in corresponding bins between the two samples correlate well (mean absolute error of WRVs being 0.000347, see Fig. 2A). Because coverage variation of cfDNA from plasma has much wider distribution than standard WGS, a sample from other plasma is more suitable than the same trio maternal sample (see Fig. 2B) for purpose of coverage distribution reference in our model. Availability of additional plasma datasets would enable us to further improve the accuracy of the reference bins.

Note that compared with previous methods, we use significantly smaller windows: ∼1 kb versus 100 kb–1 Mb used previously by Chen *et al.* (2013) and Srinivasan *et al.* (2013). As mentioned earlier, our goal here is not to detect CNVs immediately, but to rather compute a probability distribution over the number of haplotypes the fetus has inherited, which are used as priors in the more complex model. Due to the independence assumptions inherent in the HMM we want these priors, applied at each state, to be (approximately) independent, and hence we picked non-overlapping windows each containing one SNP locus.

### 2.3 HMM for CNV Inference

**Fig. 3.**
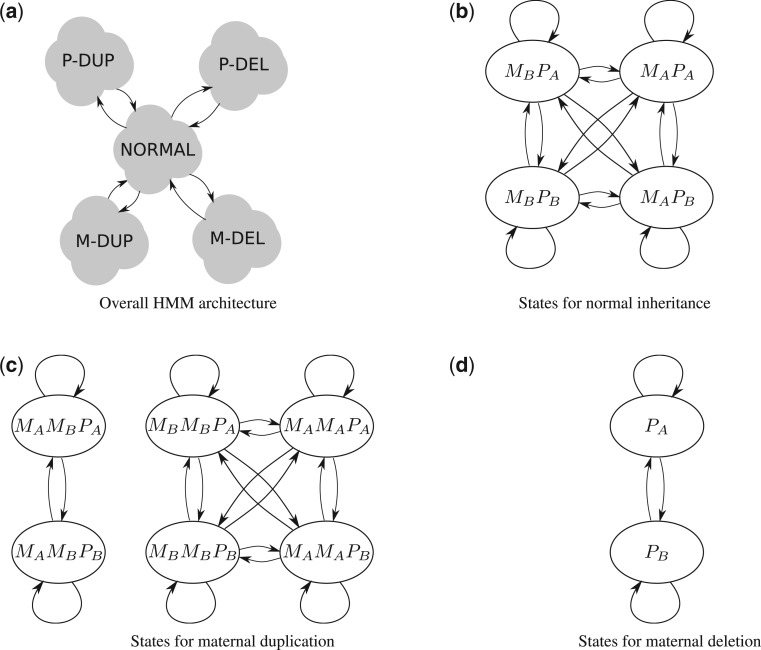
HMM used for CNV inference. (**a**) High-level architecture of the HMM with five sets of states corresponding to five types of fetal inheritance. Note, we do not allow two CNVs to be adjacent; thus, switching between two CNVs always has to go through a normal inheritance state. Edges in (a) represent edges coming in/out of all states between two sets of states. (**b–d**) Correspond to the diagram of states of the HMM within the normal inheritance, maternal duplication and maternal deletion states of (a). Paternal duplications/deletions are analogous to (c) and (d). Inner edges in (b–d) serve to model errors in phasing or recombination events

To combine the signals from individual SNP positions, we use an HMM with 20 states corresponding to modelled phased inheritance patterns (Fig. 3). That means each sate represents a possible set of parental haplotypes inherited by the fetus. States representing normal inheritance are central to the model assuming that two CNVs cannot be immediately subsequent. Between states of the same inheritance pattern, we allow for transitions reflecting either recombinations or errors in phasing. For each state, the emissions are the counts of individual alleles in reads mapped to that particular SNP position. The probability of the observed emission is the probability of such allele counts in the expected allele distribution conditional on phased inheritance pattern as described above in Section 2.1.

To incorporate the coverage information, for each SNP position, we multiply the transition probabilities into the state by the copy number priors obtained in Section 2.2. Specifically, each edge incoming to a state is multiplied by the corresponding prior of inheriting that many haplotypes, which are then normalized so that the sum of the probabilities leaving each state is one.

The transition probabilities within an event type (e.g. maternal duplication) were set to 0.01, to reflect expected haplotype block lengths of several hundred SNPs. Further, the transition probability for starting a CNV was set to one in ten thousand SNP loci (0.0001) with length expected to span approximately one thousand SNPs (i.e. transition probability back to normal inheritance was set to 0.001).

### 2.4 CNV Simulation *in silico*

To evaluate the accuracy of our CNV discovery algorithm, we created simulated datasets with CNVs of various sizes inserted into the sequenced plasma. While previous approaches have used simple Poisson modelling of the coverage of cfDNA for simulation purposes (Chen *et al.*, 2013), we propose a more elaborate model to more accurately model the extremely uneven coverage that we observe in cfDNA samples (Fig. 1). Our simulation performs the deletion or duplication of a particular fetal allele. We need to resolve the haplotypes of every individual in the trio, to correctly add or remove reads originating from a target haplotype of the CNV event. Similarly to our detection method (described in Results, below), we used Beagle 4 (Browning and Browning, 2013) with 1000 Genomes Project reference haplotypes; however, we also use the fetal genome sequenced after delivery, and utilize pedigree information to phase each individual in the trio.

To simulate a duplication, of either maternal or paternal origin, we used the parental DNA sequencing data from the family trio dataset. First, we filtered for reads mapping to the intended region of duplication that also match the target haplotype of the parent according to the parental phasing. In case of reads not uniquely mapping to either of the two parental haplotypes, i.e. the read mapped to a region without any heterozygous SNP locus, the read was selected randomly with probability 0.5. Subsequently, the filtered reads were uniformly down-sampled according to fetal DNA mixture ratio and the original plasma DOC in this region to match the expected number of reads derived from a single fetal haplotype in plasma sequencing. Resulting reads were then mixed together with original plasma reads to create a plasma sample containing the desired duplication in the fetal genome.

To simulate a deletion, we first identified a fetal haplotype inherited from the parent of choice, which was to be deleted. We filtered the plasma sample removing reads coming from this target fetal haplotype. That is, each read mapped to the intended deletion region was removed with probability of belonging to the fetus and also being inherited from the intended parent. To find this probability, we used the phasing to check which maternal and fetal haplotypes match the SNPs in the read. If none of the four haplotypes matched the read, we removed the read with probability r/2 where *r* is the fetal DNA admixture ratio. If the fetal target haplotype matched the read, it was removed with probability

(9) $$\frac{r/2}{N_{\text{m}}\cdot (1-r)/2+N_{\text{f}}\cdot r/2}$$

where $0<N_{\text{f}}\leq 2$ and $0\leq N_{\text{m}}\leq 2$ are respectively the number of fetal and maternal haplotypes that matched the read.

We also simulated plasma datasets with decreased fetal DNA mixture ratio. To achieve a desired down-rated admixture ratio r′ in our plasma sample, we had to remove appropriate number of reads coming from the fetal DNA. First, we have computed the appropriate fraction of fetal-origin reads, w.r.t. original admixture ratio *r*, to be removed from the plasma as

(10) $$r_{del}=1-\frac{1-r}{r}\cdot \frac{r\prime }{1-r\prime }$$

Similarly to simulation of a deletion, we have then filtered the plasma reads for reads originating from the fetal genome. Since this cannot be decided without ambiguity, we estimated the corresponding probability $p_{\text{f}}$:

(11) $$p_{\text{f}}(seq)=\;\{\begin{array}{cc} \frac{N_{\text{f}}\cdot r/2}{N_{\text{m}}\cdot (1-r)/2+N_{\text{f}}\cdot r/2} & \text{iff}\;N_{\text{m}}+N_{\text{f}}>0\\ r & \text{iff}\;N_{\text{m}}+N_{\text{f}}=0 \end{array}$$

where $N_{\text{f}}$ and $N_{\text{m}}$, as above, are the number of fetal and maternal haplotypes that match SNP alleles of the read. Thus a read was then removed with probability equal to

(12) $$r_{del}\cdot p_{\text{f}}(seq)$$

## 3 RESULTS

### 3.1 Datasets and processing

In our experiments, we used WGS data of two mother–father–child trios I1 (Table 1), and G1, published by Kitzman *et al.* (2012). In our experiments, we mainly used the first trio I1 with 13% fetal admixture in obtained plasma. For maternal, paternal and plasma datasets, the reads were aligned to the hg19 genome using BWA. We genotyped both the parents using Samtools and Vcftools. To improve the precision of genotyping, we only consider variants at positions previously identified as variable within the 1000 Genomes Project. Subsequently we phased the haplotypes using Beagle 4 (Browning and Browning, 2013) with reference haplotype panels from 1000 Genomes Project.

**Table 1.** Summary of mother–father–child trio I1 sequencing data [Courtesy of Kitzman *et al.* (2012)]

Individual	Sample	DOC
Mother (I1-M)	Plasma (5 ml, gestational age 18.5 weeks)	78
	Whole blood (<1 ml)	32
Father (I1-P)	Saliva	39
Child (I1-C)	Cord blood at delivery	40

### 3.2 Evaluation

We simulated 360 CNVs in I1 plasma to evaluate our method’s recall, while G1 plasma sample served as a reference in DOC-based CNV estimation as described in Section 2.2. For each test case, we picked a random position in chromosome 1, outside annotated centromere and telomeres regions, to place the simulated CNV. Our simulation methods are described in detail in Section 2.4. We then ran our algorithm on a genomic window starting 20 Mb before the simulated CNV and ending 20 Mb after the CNV. The results are shown in Table 2. We acknowledge a CNV as correctly called if CNV predictions of the same type span at least 50% of it, while precision is computed as the fraction of correct CNV calls over all calls of that category. To evaluate the effect the admixture has on accuracy, we repeated this experiment not only with the original plasma dataset, but also once down-sampled to only contain 10% admixture.

**Table 2.** Summary of recall on test set composed of 360 *in silico* simulated CNVs in I1 maternal plasma samples with 13 and 10% fetal admixture ratio

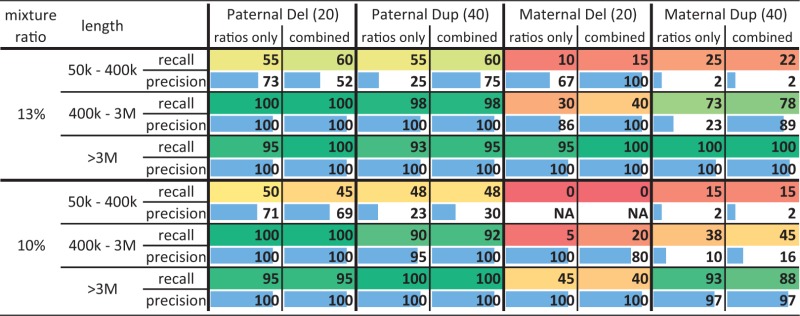

*Note*: The ‘ratios only’ column corresponds to the method that only uses allelic ratios, but not the coverage prior. In such cases both the precision and recall are mostly dominated by the model combining both signals. (We write ‘NA’ in a precision field if no call of such CNV category was predicted by the model).

The results indicate that our method can achieve nearly perfect recall and precision for variants >3 megabases, and promising results down to CNVs of 400 kb. Maternally inherited events are typically more difficult to identify than paternally inherited ones, and deletions more difficult than duplications, possibly due to complete dropout of fetal alleles due to reduced admixture.

To evaluate power of individual signals utilized by our unified model, we also tested models that take into consideration only either the allelic ratios or coverage information. The allelic ratios only model is as described above in Section 2.3 but without multiplying of copy number prior in the transition probabilities. Obtained results are shown together with the results of the unified model in Table 2.

For predicting fetal CNVs based solely on coverage information, we split the sample to bins of uniform size and computed WRVs for each, following the work of Srinivasan *et al.* (2013). We then ran a simple HMM with three states corresponding to normal inheritance, duplication and deletion, respectively. The WRVs in bins were interpreted as emissions and the emission distributions were computed as described in Section 2.2, Equation (8). We tested the HMM with bin sizes of 100 and 300 kb, and the results are summarized in Table 3. Using larger bins limit resolution of the method, e.g. in case of 300 kb bins the obtained recall on <400 kb CNVs is (close to) zero. On the other hand for large CNVs >3 Mb using 300 kb bin size mostly improves upon 100 kb bins in terms of both recall and precision.

**Table 3.** Summary of results obtained by an HMM using only WRV signal

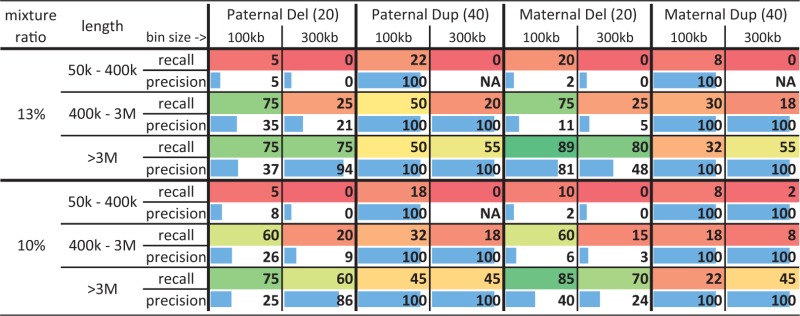

*Note*: The same test set composed of 360 *in silico* simulated CNVs was used as in Table 2. We ran the model with 100, and 300 kb bin sizes. (We write ‘NA’ in a precision field if no call of such CNV category was predicted by the model).

Note, that a direct comparison with the methods by Chen *et al.* (2013) and Srinivasan *et al.* (2013) is not possible, as they are tailored to low coverage plasma sequencing data and require a large number of control plasma samples to evaluate significance of observed coverage variation in the studied plasma sample for CNV calling.

To further test precision of our combined method, we ran our combined model on the whole plasma dataset (expected to contain no large *de novo* variants) and observed the number of CNV calls for each size. These numbers are shown in Table 4, with *in silico* accuracy for each length shown for comparison. Notably, a large fraction of the larger false-positive calls correspond to CNVs already present in parents (and hence inherited, rather than *de novo*).

**Table 4.** *In silico* recall and number of CNVs of various sizes generated in a genome-wide run

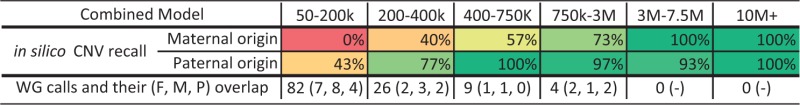

*Note*: For each CNV size, we also show (in parenthesis) the number of calls that are from at least 50% overlapped by CNVnator (Abyzov *et al.*, 2011) calls on the fetal, maternal and paternal genomes, respectively.

### 3.3 Implementation note

Our model is implemented in the Python programming language with the PyPy interpreter. When ran on a whole genome dataset, our implementation required up to 20 GB of system memory and took <4 h of single thread CPU time to finish.

## 4 DISCUSSION

In this manuscript, we introduce a novel probabilistic method for the identification of *de novo* CNVs from maternal blood plasma sequencing with largely increased sensitivity compared with methods published so far. Our method combines three types of data: allelic ratios, reflecting the changes in the expected observations of various alleles at SNP positions in the presence of the CNV; phasing information, allowing for the combining of allelic ratios across multiple SNP positions, thus improving the signal-to-noise ratio; and depth of coverage information reflecting the change in expected sequencing depth in the presence of the CNV. We apply the resulting method to simulated sequencing data, demonstrating promising results for CNVs >400 kb in length, and especially for CNVs of paternal origin.

Simultaneously, we believe our method can be further improved in several ways. First, our approach of modelling the depth of coverage prior using small windows is likely suboptimal. Especially because the method is searching for larger CNVs, using larger windows would be advantageous; however, in this case the observations of coverage at adjacent SNPs would no longer be independent, and thus not properly modelled as an HMM. We believe a more expressive model that is able to model such interactions between coverage terms would improve on the current results. Secondly, our method does not directly model potential inherited CNVs in the father (maternally inherited CNVs are modelled through the use of maternal priors at each position). Explicitly pre-computing and utilizing information about these inherited CNVs is likely to reduce the false-positive rate of ours and related methods. Thirdly, we incorporated the coverage signal in our model by comparing the observed WRV with the corresponding WRV in a reference plasma sample (G1 in our experiments). Using multiple plasma references would reduce individual-specific biases, thus improve the overall performance.

The main limitation of our method in practice is the need for deep maternal plasma cfDNA sequencing to exploit the allelic ratios signal. Note that the parental genome WGS could be replaced by genotyping using SNP arrays; however, the need for a paternal sample is a limitation for broad clinical use.
